# Optimizing Education: A Mixed Methods Approach Oriented to Teaching Personal and Social Responsibility (TPSR)

**DOI:** 10.3389/fpsyg.2019.01439

**Published:** 2019-06-28

**Authors:** Oleguer Camerino, Alfonso Valero-Valenzuela, Queralt Prat, David Manzano Sánchez, Marta Castañer

**Affiliations:** ^1^National Institute of Physical Education of Catalonia (INEFC), University of Lleida (UdL), Lleida, Spain; ^2^Biomedical Research Institute of Lleida (IRBLleida), Lleida, Spain; ^3^Faculty of Sport Sciences, University of Murcia, Murcia, Spain; ^4^Grupo de Investigación en Salud, Actividad Física y Educación (SAFE), Universidad de Murcia, Murcia, Spain

**Keywords:** teaching strategies, observational analysis, integration methods, T-pattern detection, polar coordinate analysis

## Abstract

This methodological article provides a Mixed Method approach to analyze how the Teaching Personal and Social Responsibility (TPSR) Model is feasible to enhance student’s autonomy. The objective is to detect how teachers’ behavior-oriented patterns shift in response to continuing professional development to reinforce TPSR strategies. We compared the application of TPSR by three teachers who had previously attended a training course for this model, with that of an expert in the model. A total of 44 sessions of primary and secondary school semesters in various subjects, taught by all four teachers and comprising 120 students. A mixed-method approach followed in the study involved: (a) the Observational System of Teaching Oriented Responsibility (OSTOR), which revealed how the teachers’ behavior patterns shifted over their interventions, and (b) the Tool for Assessing Responsibility-Based Education (TARE 2.0.), which focused on perceived behaviors by teachers and student behaviors. Data analysis was conducted for (a) the T-pattern detection technique, (b) polar coordinate analysis to obtain detailed sequences of instruction, and (c) descriptive and correlational analysis from the TARE. The mixed-method analysis of data confirms how the TPSR improved the teaching behaviors of the three teachers in training compared with the expert teacher.

## Introduction

Innovation in pedagogy has been shaped by great paradigms and educational perspectives moving through diverse theories such as the maturational and sociocultural theories that promote the educational involvement of social and cultural agents (Bandura, 1977; Vygotsky, 1978). In order to develop them, however, educational methods range widely from the direct instruction method, where the teacher is the main axis, to constructivist principles or scaffolding, where children build their own learning. Alongside educational methods, various teaching procedures and strategies relating to learning and teaching styles, such as problem-solving, have been deployed (i.e., Kluge, 2008). In sum, paradigms, theories, methods, procedures, and strategies are always linked in order to convey the teacher’s style (Castañer et al., 2010, 2013a; Waring and Evans, 2015). In this study we focus on the Teaching Personal and Social Responsibility model (TPSR, Hellison, 1978, 1985) as a pedagogical model that enhance personal and social responsibility.

### Enhance Personal and Social Responsibility

The Teaching Personal and Social Responsibility model (TPSR, Hellison, 1978) is a curriculum and pedagogical model based on the assumption that students need to learn to be responsible for themselves and others in order to socially interact in a suitable way (Hellison, 1985), and this is a goal that is implicitly included in current Spanish legislation. “One of the principles on which the Spanish Educational System is based on the transmission and implementation of values that favor personal freedom, responsibility, democratic citizenship, solidarity, tolerance, equality, respect, and justice” (LOMCE, 2013). The TPSR model-based program suggests five levels of responsibility: (1) respect for the rights and feelings of others; (2) self-motivation; (3) self-direction; (4) caring; and (5) transfer “outside of the gym” (Hellison, 2011). Moreover, the TPSR model-based program provides a specific lesson plan format, as well as teaching strategies to support the implementation program, which teachers adapt to their context.

This model is regarded as one of the most effective approaches in terms of developing values in adolescence, given the positive results it has achieved (Escartí et al., 2010a). It has been applied in numerous studies which relate it to improvements in responsibility levels (Hellison and Wright, 2003), self-efficacy levels (Escartí et al., 2010a), cognitive, participation, and relatedness improvements (Likfa, 1990), self-control and sportiness (Cecchini et al., 2007), cognitive improvement (DeBusk and Hellison, 1989), and interpersonal skills (Cutforth and Puckett, 1999), as well as to better grades and lower levels of absenteeism (Wright et al., 2010). Furthermore, life satisfaction and lower academic stress are strongly related to personal responsibility levels and to academic performance (Smithikrai, 2013).

Some of these variables (sportsmanship, violence, and personal and social responsibility) have been linked in studies in PE classes and in school sports, showing how encouraging sportsmanship or personal and social responsibility can prevent violent behaviors (Sáenz et al., 2012). Several studies have demonstrated that although teachers’ adherence to the TPSR model was deemed moderate, the strategies they used to foster responsibility were significantly correlated with students’ increasingly responsible behaviors (Escartí et al., 2018; Sánchez-Alcaraz et al., 2019). This evidence points to the need for more in-depth educational research in this line of action. In this study we apply a mixed-method approach involving two specific techniques: T-pattern detection, to detect repeated behavioral patterns, and polar coordinate analysis, to detect significant associations between teaching behaviors in various curricular subjects.

### Professional Development for Teachers

Continuing professional development (CPD) for teachers is considered crucial for moving away from traditionally dominant pedagogical practices, such as PE practices, to meet the needs of contemporary students (Armour et al., 2017). Unfortunately, there is no clear evidence of an effective form of CPD. Moreover, pedagogical changes among teachers are considered to be evidence-based and dependent upon teachers’ understanding of student responses to their instructional approach (Goodyear et al., 2014). Sadly, this is no easy task and demands complex research projects to connect teacher practice and student learning. However, there has been a call to assess the impact of sustained school-based CPD on teacher practices and student learning to gain new insights into the characteristics of effective programs.

Due to the importance of the TPSR as one of the best models for promoting values, responsibility, and life skills, several studies (Pozo et al., 2016) place particular importance on future research going forward in two directions: (a) the TPSR application requires surveillance and professional assessment; and (b) longitudinal studies with follow-up data and *ad hoc* methodological designs. This study takes into account the first direction because the TPSR model emphasizes a strong teacher-student relationship, and throughout the teaching process of the study teachers followed a CPD (Hemphill et al., 2015). Moreover, this study takes into account the second direction because it has implemented a mixed-method design (Anguera et al., 2012, 2014, 2017; Camerino et al., 2012; Castañer et al., 2012, 2013b) that merges quantitative and qualitative data using various methodological tools and techniques.

As the purpose of this research project is to fulfill the need of more in-depth educational research on TPSR and to broaden the knowledge of its effects, the objective of this study was to detect how teachers’ behavior-oriented patterns shifted in response to CPD to reinforce TPSR strategies.

## Materials and Methods

### Research Design

Although current pedagogic discourse points out the importance of integrating qualitative and quantitative data using mixed methods research (Creswell, 2003, 2015; O’Cathain et al., 2010), numerous researchers still struggle to merge the two approaches and restrict their research to instruments (i.e., only questionnaires) and data (only quantitative or qualitative) of the same etiology. Fortunately, in the last decade some researchers of pedagogical models—and more specifically the TPSR model—have implemented mixed methods approaches, for example, within the PE context (Escartí et al., 2010b; Gordon, 2010; Hemphill et al., 2015). Thus, by incorporating observational methodology, we also used a mixed methods approach because we had already demonstrated its effectiveness in previous research (Camerino et al., 2012; Castañer et al., 2012, 2016a; Anguera et al., 2014, 2017; Casarrubea et al., 2018).

There is a lack of observational methodology in research relating to TPSR. Therefore, we used systematic observational methodology (Anguera, 2003), which has proven to be effective in teacher strategies and communication analysis (Castañer et al., 2010, 2012, 2013a, 2016b; Alves Franco et al., 2013; Torrents et al., 2013), combined with perceived behaviors by teachers themselves. T-pattern detection and polar coordinate analysis exemplify the most powerful specific techniques of observational methodology which has proven to be effective in previous research (Castañer et al., 2011; Lozano et al., 2016; Fernández-Hermógenes et al., 2017) and could provide essential input on pedagogical research.

### Participants

Overall context: the study involved two different schools (one primary and one secondary schools) from a Spanish region with a similar low and middle-level socio-demographic profiles.

Teachers. Four teachers with a similar level of experience in the national educational system (between 5 and 10 year teaching in their subjects) were analyzed, who were labeled as follows:

Teacher 1, PE teacher in the first stage of secondary education (2 lessons per week for 55 min).

Teacher 2, History teacher in the first stage of secondary education (4 lessons per week for 55 min).

Teacher 3, Spanish language teacher in the final stage of primary education (4 lessons per week for 55 min).

Teacher 4, PE teacher in the final stage of primary education (2 lessons per week for 55 min).

The contents developed for each teacher (Table 1) in the different subjects where those included in the current Spanish Educational System (LOMCE, 2013). All teachers reached at least the first level of responsibility in lesson 5 and the second level in lesson 10. Teachers 1 and 4 implemented all their lessons in an indoor gym and outdoor courts. Teachers 2 and 3, implemented all their lessons in a usual classroom and a computer room. Teachers 1, 2, and 3 had been trained in TPSR and were unaware of the TPSR methodology (inexperienced teachers in TPSR). Teacher 4 was familiar with the TPSR methodology thanks to an initial training course and 3 year experience (experienced teacher in TPSR).

**Table 1:** Lessons, responsibility levels, strategies, contents, and task examples among the implementation.

Number lesson	Responsibility level and Strategies	PE secondary Teacher 1	History secondary Teacher 2	Spanish language primary Teacher 3	PE primary Teacher 4
1–5	L1 and L5 Introduction to TPSR, responsibility contracts, cooperative activities, conflict resolution	Fitness: tests, strength, endurance, speed and mobility	Prehistory: paleolithic, neolithic and metal age	Vocabulary: types of dictionary Spelling: accentuation rules Grammar: text, paragraph, sentence and word	Cooperative-Opposition games
6–10	L2 and L5 (reinforce L1) Cooperative challenge tasks	Latin dancing: salsa and merengue	Old Age: Egypt, Greece and Rome	Vocabulary: synonyms and antonyms Spelling: accentuation Grammar: Syntax	Volleyball: technique and tactic Physical condition: test and comparison of outcomes
Task example Level 1		Circuit training: in groups of 4–5 people. They have to do a number of repetitions in every station, student may do them or not but at least they have to go together.	Historic timeline: in groups 4–5 people. They have to draw a timeline with the events that occurred during the studied periods, giving to the students the choice not to participate but respecting the rest of the mates.	Literature: in small groups of 5 people, read the book “The Little Prince”. Every student has to write the character with he/she feels more represented, making a story among all of them and telling the rest of the groups. Those who do not want to participate can only write their character.	Dodge ball game, with two fields and three cemeteries. Students who do not want to play, can be settled in the central cemetery to retrieve balls that go out and leave them in the center to be taken by the fastest player.
Task example Level 2		Creating a choreography: students have to create a merengue choreography where everyone has to contribute with an individual step and participate in the group choreography.	Punic Wars: from an event list, students have to answer as many question as they can individually.	Syntax: each Student receives a list with 10 syntax problems, in progression of complexity. Each Student tries to solve all that he/she can, receiving a point or a reward for each sentence he/she gets to do rightly.	A volleyball reduced game: they have to play a 4vs4 match and they have in a Borg scale (1–10) to up 8 points.
Task example Level 3		^∗^Fitness: in small groups of 4 students, have to expose to the rest of the classmates a progression routine to improve the speed, strength or endurance.	The Great Battles. Students have to do an individual task where they look for information about an history battle, origin, main characters, current consequences and personal conclusions to expose at the end of the learning unit to the classmates.	^∗^Spelling: accentuation rules. Individually, each student has to look for on internet typical words from Murcia Region, indicating if they have the stress in the final, second-to-last or third-to-last syllable. Verbal explanation to classmates of the meaning of these words.	Individual work plan. Students after doing Alpha Fitness Children Battery and comparing their outcomes with the average values, they will elaborate an individual work plan with 5 sessions to improve the physical ability they most like and with that with the lowest outcome.
Task example Level 4		^∗^Fitness: groups of 5 students have to create their own circuit training with 4 stations to improve their strength. One student of the group will be responsible for choosing the next station to go and finally, he/she explains to the rest of the groups what they have done in every one of the four stations.	^∗^History of Rome: groups of 4 students, each group does its own work on the History of rome for 5 lessons. Each lesson will have a leader who will be responsible for writing the report to be delivered to the teacher at the end of each class.	^∗^Spelling: groups of 5 students play the contest “Up the pencil”. The teacher says a letter and a family or words (for example A and fruits). Each group collects as many words as possible and the leader of every group chooses only those ones which are right. When the teacher gives the final sign every leader will say all the words of his/her group had collected.	^∗^Cooperative/opposition games: groups of 4 students have to play an alternative games (for example “colpbol”. The skillest players will help the rest of the team to get a goal.
Task example Level 5		^∗^Latin dancing: workshop for famílies. Students teach a latin dance choreography to their parents, including some steps they have learnt previously during the physical education lessons.	^∗^Ancient world: An ancient theater. Students are invited to participate in a theater play about Punic Wars where, they can choose between being audience or actors and actresses.	^∗^Accentuation: after working accentuation and grammar rules, the game “goose of the letters” is carried out, inviting the 4th grade students, playing a human goos in teams, with 4th level Language qüestions. Each 4th grade Student is accompanied by a 6th grade Student who help him but never say the answer.	^∗^Cooperative games: the 6th grade students after finishing the cooperative games unit, in the party at the end of the term, they invite the 4th grade students to participate in a game session lead for them.

*Note: L1, L2, and L5: Responsibility levels.*

Students. The study also involved 120 students (57 females and 63 males) aged between 11 and 16 (*M* = 13.8 years, *SD* = 2.3), who were taught by the four participant teachers, no one of them had previous experience with TPSR and were selected based on accessibility and convenience. For student age selection, as a point of interest we included the first stage of secondary education, defined according to current legislation in Spain (LOMCE, 2013), along with the final year of primary education, which would mark the boundary between the penultimate and final stages of Piaget’s cognitive development (Piaget and Cook, 1952).

The study was carried out in accordance with the Declaration of Helsinki and was accepted and verified by the Ethics Committee of the University of Murcia, Spain (ID 1685/2017). As the observational methodology we use is always conducted over natural context, we observed the natural development of the scholar sessions, therefore written informed parental consent was not obtained for the purposes of research participation and was not required as per applicable institutional and national guidelines. Regarding video recording, the institution has a consent form about image privacy that parents of students enrolling at the school are required to sign. All four teachers signed a consent form in order to participate in the research study, the consent obtained was both written and informed.

### Materials

A mixed methods approach was followed in this study: (a) the Observational System of Teaching Oriented Responsibility (OSTOR) adapted from the Spanish version SORPS (Prat et al., 2019a,b) was used to obtain teacher behavior patterns and (b) the Tool for Assessing Responsibility-Based Education 2.0. (TARE 2.0, Escartí et al., 2015) focused on perceived behaviors of teachers and students by two external observers based on the Tool for Assessing Responsibility-Based Education (TARE, Wright and Craig, 2011).

#### Observational System of Teaching Oriented Responsibility

The OSTOR (Table 2) comprised six criteria. Four criteria related to teacher actions: (1) (Expectations); (2) (Explanations); (3) (Organization); (4) (Task adjustments). One criterion related to the student: (5) (Student’s responses). And a final criterion related to the last session: (6) (Session summary). Each criterion was expanded to build an exhaustive and mutually exclusive observation system that included a total of 18 categories.

**Table 2:** Observational System of Teaching Oriented Responsibility *(OSTOR)*.

Criterion	Category	Code	Description
Expectations	Objective of Session	OBS	Prospects and aims of the session
	Objective of Task	OBT	Prospects and aims of the task
Explanations	Imposition Instructions	IMP	Without the possibility to include changes
	Shared	SHA	Proposals are allowed to be decided in common
Organization	Established	EST	Spaces and materials are mandated
	Distribution of Function	DIS	Functions and roles are allocated
	Suggested	SUG	Teachers pose opportunities to pupil’s interventions
Task adjustments	Negative Evaluation	NEG	Rebuke to the students
	Redirect	RED	Correct student’s responses
	Positive Evaluation	POS	Encourage and motivate the students
	Proposals	PRO	Formulate new options to be successful
Student’s responses	Reproduction	REP	Replicate tasks or situations
	Unbalances	UNB	Disarranged or disordered responses
	Autonomy and Leadership	AUT	Drive initiatives
	Self-Assessment	SAS	The student evaluates its own performance
Session summary	Guided Summary	GUS	The teacher summarizes the session
	Shared Summary	SHU	The students take part to the sessions summary
	Nonexistent Summary	NSU	The sessions end without be summarized

#### Recording Instrument *(LINCE)*

For recording teaching behavior sequences, sessions were coded using the free instrument software LINCE (v.1.2.1) (Gabin et al., 2012) and LINCE PLUS (Soto et al., 2019). This software program was also used for the data quality check. LINCE has been designed to facilitate the systematic observation of spontaneous behaviors in any situation or habitual context. LINCE is highly practical and easy to use, and integrates a wide range of functions: coding, recording, obtaining data quality, and enabling data export to several data analysis applications. The exported LINCE information was analyzed using two programs for data analysis: (a) THEME software package (Magnusson et al., 2016) for T-pattern detection; (b) HOISAN v.1.6.3.2 (Hernández-Mendo et al., 2014) for polar coordinate analysis.

#### Assessing Responsibility-Based Education *(TARE)*

Assessing Responsibility-Based Education 2.0. (TARE) by Escartí et al. (2015) was used to obtain teacher and student behaviors coding 3-min intervals. This instrument has already been applied in previous studies (Escartí et al., 2015; Ivy et al., 2018) and has a Likert scale: 0 (Absent), 1 (Weak), 2 (Moderate), 3 (Strong), and 4 (Very strong), and consists of a two-part observation scale:

Student responsibility: (1) Participation: the student is “on task,” i.e., following directions and participating in activities or tasks organized by the teacher. (2) Engagement: the student seems to have a high level of interest and motivation for the task or for the educational activity that is evident in their level of active contribution. (3) Showing Respect: the student is actively showing respect for others, i.e., making eye contact, paying attention to others, or actively listening. (4) Cooperation: the student demonstrates the social skills needed to work effectively with others in accomplishing a common task. (5) Encouraging Others: the student offers social support to others in proactive ways. (6) Helping Others: the student takes on helping roles. (7) Leading: the student takes on a leadership role with regard to an educational task. (8) Expressing Voice: the student makes suggestions, shares opinions, and/or reflections in ways that express their personality and individuality. (9) Asking for Help: the student seeks out assistance and asks for help from the teacher or peers.

Teacher responsibility: (1) Modeling respect means the teacher models respectful communication. (2) Setting Expectations means the teacher explains or refers to explicit behavioral expectations. (3) Opportunities for Success means the teacher structures the lesson so that all the students have the opportunity to successfully participate and be included, regardless of individual differences. (4) Fostering Social Interaction means the teacher structures activities that foster positive social interaction. (5) Assigning Management Tasks means the teacher assigns specific responsibilities or management-related tasks that facilitate the organization of the program or a specific activity. (6) Leadership means the teacher allows students to lead or be in charge of a group. (7) Giving Choices and Voices means the teacher gives students a voice in the program. (8) Role in Assessment means the teacher allows students to have a role in learner assessment. (9) Transfer means the teacher directly addresses the transfer of life skills or responsibilities from the lesson to contexts beyond the program.

### Procedure

#### Recording Procedure

A camera was installed in the classroom six sessions prior to commencement to familiarize students and avoid non-spontaneous behaviors. An initial session of all four teachers, pre-intervention session (the experienced teacher in TPSR and the inexperienced ones) was registered and coded. The three inexperienced teachers then undertook a TPSR course based on an intensive teacher training process. After training, one weekly session of all four teachers was registered and coded (44 sessions, 11 for each teacher) over a 2-month period. In addition, the research team assessed the teachers on a weekly basis, giving feedback through a written online document and in a one-to-one meeting, and providing suggestions for improved model implementation from the results obtained. Teaching behavior sequences were analyzed from the moment the teacher effectively started the session, that is, disregarding the time spent checking the attendance list.

For appropriate training in using OSTOR, before the full data set was coded, two expert observers recorded one session per teacher, which was not included in the final sample. Intra- and inter-observer reliability was calculated from that session in LINCE, resulting in a kappa statistic of 0.95 for inter-observer and 0.98 for intra-observer analysis.

#### Specific Teacher Training

The correct implementation of any program requires specific teacher training (Lee and Choi, 2015). Inexperienced teachers were trained in TPSR in a two-phase approach:

1. A 10-h course on TPSR theory and practice: a group of 29 teachers in Primary and Secondary school were instructed how to design classroom climates according to the model-based program, and were provided with global and specific strategies for the development of responsibility in PE. Firstly, they received the theoretical foundations of TPSR Model, the lesson structure, the five different levels of responsibility, the general strategies and specific strategies for teaching responsibility, the strategies for solving problems. Secondly, the teachers acted like students in a practical lesson based on the TPSR Model. Teachers were splitted up in two groups, one of them was made up of physical education teachers (12 teachers) and implemented a practical lesson in a sports court. The other group (17 teachers) was made up of those teachers who taught other subjects such as Mathematics, Literature, Spanish Language, Historic, etc., and implemented a lesson in a classroom. The main changes done in the group of teachers in the classroom were: (i) part 3 and 4 of the lesson structure were jointed, and (ii) some new strategies were incorporated to improve teaching responsibility levels in the classroom. For example, to promote level 2, the “petals blackboard” strategy was created, where a flower without petals is drawn on the blackboard and students must complete the class activities to achieve the petals, making a count at the end of the lesson to show the values of participation and effort reached. Eventually, their knowledge was assessed both with a multiple choice questionnaire and by completing a lesson implementing in their subject.
2. Continuous training: three teachers who were interested in following the TPSR study, had signed a consent form and their respective schools had a consent form signed about image privacy of students and were enrolled throughout the 2-month program, the main researcher met with them before and during implementation. Beforehand, the teachers outlined the sessions they planned to carry out with the responsibility strategies; the main researcher then assessed the session and provided appropriate (or correcting or guiding) feedback.

The goal was to develop a class climate to promote responsibility through the application of TPSR. Students learned responsibility progressively, moving through the different levels (Escartí et al., 2013). Each session format followed Hellison’s (2011) five-part proposal: (1) Relational time; (2) Awareness talk; (3) Physical activity plan; (4) Group meeting; and (5) Reflection time (for students to self-assess their own responsibility). Teachers used both general strategies (e.g., being an example of respect, setting expectations, providing opportunities for success) and specific strategies (e.g., redefining success, personal work plans, responsibility for students of other groups) to implement TPSR. They also used strategies to solve individual conflicts (e.g., progressive separation from the group) and collective conflicts (e.g., accordion principle) thereby fully integrating TPSR in their PE classes (Escartí et al., 2013). In addition, at the end of each session, teachers also had to self-assess their performance using the TARE (Wright and Craig, 2011) to encourage reflection on the implementation of the model-based program, answering in a dichotomous (yes/no) scale.

#### Fidelity of Implementation

Hastie and Casey (2014: p. 423) believe that researchers need to provide: (a) a rich description of the curricular elements of the unit, (b) a detailed validation of model-based program implementation, and (c) a detailed description of the “program context” for readers to acquire an exact and comprehensive understanding of the research design and the outcomes obtained. Parts (a) and (b) have already been detailed in the “Specific teacher training” section. For a detailed validation of model implementation, the research team videotaped one session every week (40 lessons), apart from an initial session before the implementation program.

These video recorded lessons were analyzed independently by two external observers using the TARE 2.0 instrument (described in the “Assessing Responsibility-Based Education TARE” section). They were two university researchers who had more than 5 years of experience in this kind of analysis and were trained following the sequence established by Wright and Craig (2011). First, explanation and clarification of the meaning of each of the categories of the tool (they were put in different situation examples to distinguish them clearly). Second, the observers together watched two complete classes implementing the TPSR (corresponding to a two lessons applied in a different school not related to the present study) using TARE 2.0. Third, the results of the observers were shared to unify criteria. Fourth, when observers obtained an inter-reliability of 80%, we took such inter-reliability to be satisfactory, thus that the observers were ready to start the analysis of the study sessions.

### Data Analysis

From the 44 sessions that were systematically observed, the pre-intervention (*k* = 4) and final sessions (*k* = 4) of each teacher were analyzed to obtain teacher behavior patterns using OSTOR and TARE 2.0 instruments (124 × 3-min intervals). On the other hand, to get to know the strategies used by teachers to promote responsibility and the differences between subject areas, all the TPSR intervention sessions (40 lessons/632 × 3-min intervals) using TARE 2.0 were analyzed.

Data analysis was conducted using two particularly fitting techniques for analyzing such complex teaching behaviors in order to obtain detailed sequences of instruction: (a) T-pattern detection and (b) polar coordinate analysis. Both techniques pinpointed synergies in terms of the behaviors obtained. The differences over the implementation program and between teacher behavior and student responses subject areas were analyzed by means of TARE 2.0.

#### T-Pattern Detection

T-pattern detection (temporal pattern detection) (Casarrubea et al., 2015; Magnusson et al., 2016) is a relevant data analysis technique in systematic observation. The function of T-pattern analysis is to detect repeated behavioral patterns that are invisible to unaided observers because the temporal structure of complex behavioral sequences is composed of observable event-types (Magnusson et al., 2016). THEME software is a powerful research tool for obtaining T-patterns using an evolution algorithm which compares all patterns and retains only the most complete. Because any basic time unit can be used, behavioral structures can be explored in detail and stronger connections between successive recorded behaviors are revealed. T-pattern detection has been successfully used in several research fields (Burgoon et al., 2016; Pérez-Tejera et al., 2018) to reveal hidden behaviors and underlying pedagogical actions (Castañer et al., 2010, 2013a; 2016b; Rodríguez-Dorta and Borges, 2017; Prat et al., 2019a). In sum, T-pattern detection is an analysis technique that scrutinizes all coded behaviors and their combinations, revealing which ones establish a behavior pattern that appears several times throughout the observed sessions. THEME software (Magnusson et al., 2016) detects T-patterns from the most to the least complex in relation to the number of branches (dendrogram diagram) that comprise the pattern.

#### Polar Coordinate Analysis

Polar coordinate analysis was developed by Sackett (1980) and later improved by Anguera (1997). It involves the detection of significant associations between a focal behavior (the behavior of interest) and conditional behaviors (the other behaviors analyzed). Polar coordinate analysis provides a vectorial representation of the complex network of interrelations between carefully chosen, exhaustive, and mutually exclusive defined criteria of behaviors. It is a powerful data reduction technique that is increasingly being used in studies (Castañer et al., 2016a, 2017; López Jiménez et al., 2016; Arias-Pujol and Anguera, 2017).

As stated in previous research (Castañer et al., 2016a), Figure 1 gives a graphical explanation of how to interpret the associations between the focal behavior (F), placed in the center of the figure, and the conditional behaviors in each quadrant. The association is shown both quantitatively (length of vector) and qualitatively in quadrant I, II, III, or IV, as follows:

**FIGURE 1:**
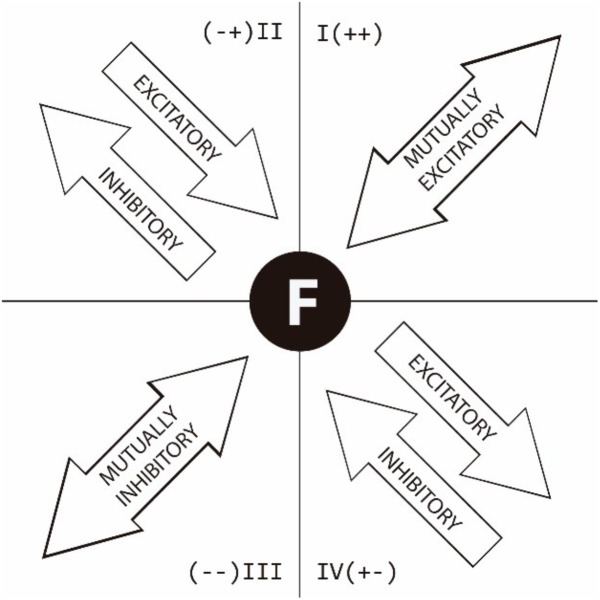
Graphical explanation of how to interpret polar coordinate quadrants (Castañer et al., 2016a, p. 5).

Quadrant I (++). The given and conditional behaviors are mutually excitatory.

Quadrant II (− +). The given behavior is inhibitory and the conditional behavior is excitatory.

Quadrant III (− −). The given and conditional behaviors are mutually inhibitory.

Quadrant IV (+ −). The given behavior is excitatory and the conditional behavior is inhibitory.

#### Data Analysis From TARE

TARE 2.0 offers descriptive statistics analysis conducted to obtain the values of teacher intervention and student responses. In turn, these values provided an assessment of the extent to which teachers promoted responsibility throughout their lessons. Inter-reliability was obtained using the agreements/(agreements + disagreements) × 100 (García-López et al., 2012). The total agreement for teacher behaviors was 84.7% and for student behaviors 82.8% before starting the analysis of the lesson in the present study.

A descriptive analysis of the TPSR strategies used by teachers to promote responsibility was carried out with the 40 implemented lessons (632 × 3-min intervals). Normal distribution was verified using the Shapiro Wilk test (*p* > 0.05), before applying T test for related samples to obtain differences between pre-intervention and last lessons of the TPSR implementation for each teacher (between 14 and 16 × 3-min intervals per teacher in the pre-intervention lessons and 15 and 17 × 3-min intervals in the last lessons). Finally, after verifying the normal distribution with the Kolmogorov-Smirnov (*p* > 0.05), a T test for independent samples was then conducted for each strategy to contrast the results between the different subject areas (316 × 3-min intervals of physical education lessons versus 316 × 3-min intervals of other subjects). The software used for the analysis was IBM SSPS 22.0.

## Results

### Strategies Used by Teachers to Promote Responsibility

To evaluate the instruction and treatment validity, the use of strategies to promote responsibility, the Likert scale value (0–4) of the nine teacher categories measured by the TARE 2.0 was assessed (Table 3). The descriptive analysis reflected values above zero in all the variables studied. The mean every 3-min intervals for each strategy was always above zero and greater than one except for the strategies ‘transfer’ and ‘leadership’ of participant 3.

**Table 3:** Teachers’ Strategies used to Promote Responsibility.

	Teacher 1 *M (SD)*	Teacher 2 *M (SD)*	Teacher 3 *M (SD)*	Teacher 4 *M (SD)*
The teacher….				
Respect model	4.00 (0.00)	3.91 (0.28)	3.16 (0.49)	3.98 (0.07)
Expectations	2.39 (0.72)	2.27 (0.74)	3.09 (0.75)	2.60 (0.80)
Opportunities	2.14 (0.79)	2.16 (0.75)	2.39 (0.80)	2.60 (0.55)
Interaction	1.88 (0.63)	3.03 (0.82)	2.26 (1.18)	2.38 (0.94)
Assigning tasks	2.29 (0.77)	2.56 (0.96)	2.31 (1.09)	2.28 (0.57)
Leadership	2.45 (0.98)	1.21 (1.16)	0.52 (0.97)	2.77 (0.75)
Giving choices	1.79 (0.69)	2.92 (0.49)	2.35 (1.38)	2.30 (0.54)
Assessment	1.06 (1.06)	1.08 (0.93)	1.70 (0.95)	1.37 (1.25)
Transfer	0.17 (0.17)	0.49 (0.70)	0.53 (0.55)	0.45 (0.55)

*M = Mean. SD = Standard Deviation.*

### Initial and Final T-Patterns Detected

**FIGURE 2:**
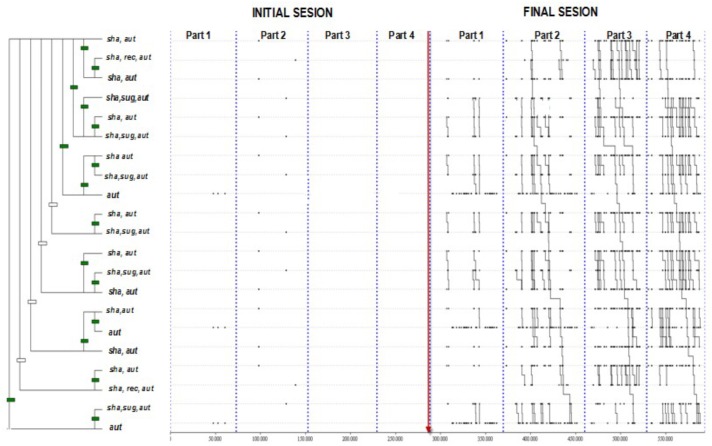
T-pattern from the four initial and final sessions of each teacher.

From the total of T-patterns detected, we selected a common T-pattern obtained from the four initial and four final sessions of each teacher (Figure 2). This common T-pattern is relevant because all sets of behaviors that comprise the 21 branches of the T-pattern tree include student behavior of Autonomy (AUT). Some of these sets also contain Shared (SHA) and Suggestions (SUG) associated with Autonomy (AUT). The left side of Figure 2 shows the practical nonexistence of those behaviors in the four initial sessions of all teachers, including the experienced teacher (teacher 4) (pre-intervention). Those initial sessions were compared with the four final sessions which show many of those behaviors, as seen on the right side of the Figure 2.

### Mixing T-Patterns and Polar Coordinates

**FIGURE 3:**
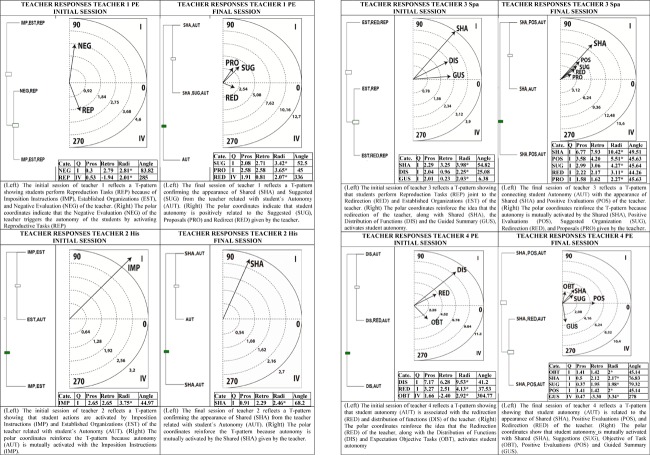
T-pattern and polar coordinate data of both the initial and final sessions of the teachers.

Because our methodological aim is a mixed-method approach, we decided to offer a new graphical depiction that clearly connects and contrasts T-pattern and polar coordinate data of both the initial and final sessions of the teachers (Figure 3). We selected a total of eight images for this new understanding of data contrast from the T-pattern and polar coordinate connection. The left side of the images includes a T-pattern related to the polar coordinate obtained, which in turn appears on the right side of the image.

All polar coordinate analysis was conducted taking the behavior of Autonomy (AUT) as the focal behavior, because it is an essential part of the TPSR model. Furthermore, the autonomy of the students gained relevance in the common T-pattern shown in Figure 2.

### TARE Results

#### Contrasting Pre-intervention and Final Sessions

The results of TARE 2.0, obtained from the two observers (Table 4), show the differences between the variables of the pre-intervention session (first rows), before commencement of TPSR, and the final session (second rows), using TPSR. The categories differentiate both types of participants: teacher and student.

**Table 4:** Results of TARE 2.0 in teacher and student behaviors.

		Teacher 1		Teacher 2		Teacher 3		Teacher 4	
Category	Variable	*M (SD)*	*p*	*M (SD)*	*p*	*M (SD)*	*p*	*M (SD)*	*p*
Teacher	Respect model	4.00 (0.00) 4.00 (0.00)		4.00 (0.00)		2.89 (0.68)		4.00 (0.00)	
			1.000	4.00 (0.00)	1.000	3.63 (0.50)	0.001ˆ***	4.00 (0.00)	1.000
	Expectations	0.00 (0.00)		0.00 (0.00)		0.50 (0.92)		2.50 (1.03)	
		3.08 (1.08)	0.001^∗∗∗^	2.60 (1.06)	0.001^∗∗∗^	3.63 (0.89)	0.001^∗∗∗^	1.76 (1.09)	0.056
	Opportunities	0.00 (0.00)		0.00 (0.00)		1.33 (1.28)		2.81 (0.75)	
		2.75 (1.36)	0.001^∗∗∗^	2.53 (1.06)	0.001^∗∗∗^	3.69 (0.87)	0.001^∗∗∗^	2.65 (0.93)	0.580
	Interaction	0.00 (0.00)		0.57 (0.51)		1.39 (1.33)		3.00 (0.97)	
		2.33 (0.89)	0.001^∗∗∗^	3.33 (1.40)	0.001^∗∗∗^	3.25 (1.18)	0.001^∗∗∗^	2.12 (0.33)	0.001^∗∗∗^
	Assigning tasks	2.75 (1.91)		0.00 (0.00)		2.61 (1.82)		2.19 (1.28)	
		1.33 (0.98)	0.028^∗^	2.27 (1.16)	0.001^∗∗∗^	2.75 (1.57)	0.814	2.65 (.93)	0.244
	Leadership	0.00 (0.00)		0.00 (0.00)		0.00 (0.00)		2.94 (1.39)	
		2.50 (1.88)	0.001^∗∗∗^	1.13 (0.74)	0.001^∗∗∗^	0.19 (0.40)	0.057	3.06 (1.75)	0.828
	Giving choices	0.75 (0.45)		0.79 (0.43)		0.50 (1.15)		2.44 (1.46)	
		2.08 (0.90)	0.001^∗∗∗^	2.47 (1.06)	0.001^∗∗∗^	3.19 (0.98)	0.001^∗∗∗^	2.00 (0.50)	0.252
	Assessment	0.00 (0.00)		0.00 (0.00)		0.00 (0.00)		1.31 (1.25)	
		2.17 (1.53)	0.001^∗∗∗^	2.47 (1.60)	0.001^∗∗∗^	3.50 (0.73)	0.001^∗∗∗^	0.12 (0.49)	0.001^∗∗∗^
	Transfer	0.00 (0.00)		0.00 (0.00)		0.00 (0.00)		0.19 (0.75)	
		0.50 (0.90)	0.082	1.27 (0.70)	0.001^∗∗∗^	1.31 (1.08)	0.002^∗∗^	0.29 (0.47)	0.626
Student	Participation	1.75 (0.93)		0.79 (0.43)		2.00 (0.00)		3.25 (0.86)	
		3.67 (0.49)	0.001^∗∗∗^	3.67 (0.72)	0.001^∗∗∗^	3.50 (0.63)	0.001^∗∗∗^	3.71 (0.47)	0.065
	Engagement	1.44 (0.81)		2.00 (0.00)		1.78 (0.65)		2.25 (0.68)	
		2.67 (0.49)	0.001^∗∗∗^	2.87 (0.35)	0.001^∗∗∗^	2.81 (0.54)	0.001^∗∗∗^	3.00 (0.00)	0.001^∗∗∗^
	Respect	1.44 (0.51)		4.00 (0.00)		3.00 (0.00)		3.19 (0.54)	
		2.92 (1.00)	0.001^∗∗∗^	4.00 (0.00)	1.000	3.00 (0.00)	1.000	3.00 (0.00)	0.165
	Cooperating	0.81 (0.83)		0.00 (0.00)		0.44 (0.86)		2.13 (1.26)	
		1.75 (1.29)	0.027^∗^	3.67 (0.72)	0.001^∗∗∗^	2.44 (1.09)	0.001^∗∗∗^	1.41 (0.94)	0.073
	Encouraging	0.50 (0.52)		0.00 (0.00)		0.00 (0.00)		1.75 (1.00)	
		1.83 (1.34)	0.001^∗∗∗^	1.73 (0.70)	0.001^∗∗∗^	1.81 (1.17)	0.001^∗∗∗^	1.41 (0.94)	0.324
	Helping	0.75 (0.86)		0.00 (0.00)		0.00 (0.00)		1.13 (1.02)	
		2.00 (1.48)	0.009^∗∗^	1.73 (0.70)	0.002^∗∗^	0.81 (0.40)	0.001^∗∗∗^	1.41 (0.94)	0.408
	Leading	0.00 (0.00)		0.00 (0.00)		0.00 (0.00)		3.13 (1.63)	
		2.83 (1.75)	0.001^∗∗∗^	1.73 (0.70)	0.002^∗∗^	0.81 (0.40)	0.001^∗∗∗^	1.41 (0.94)	0.001^∗∗∗^
	Expressing	0.00 (0.00)		0.57 (1.09)		0.00 (0.00)		2.50 (2.00)	
		3.17 (1.03)	0.001^∗∗∗^	2.87 (0.52)	0.001^∗∗∗^	3.06 (0.68)	0.001^∗∗∗^	2.59 (0.94)	0.871
	Help	0.00 (0.00)		0.00 (0.00)		0.00 (0.00)		0.13 (0.34)	
		1.50 (1.68)	0.001^∗∗∗^	0.00 (0.00)	1.000	0.63 (0.62)	0.001^∗∗∗^	0.00 (0.00)	0.141

*Note: p < 0.05 = ^∗^; p < 0.01 = ^∗∗^; p < 0.001 = ^∗∗∗^; Teacher 1 and Teacher 4 = PE teachers; Teacher 2 and Teacher 3 = History and Spanish language teachers.*

Table 4 based on the data recorded with the teacher observation section of TARE 2.0, there were statistically significant differences in most behaviors of teacher 1, 2, and 3, except in the respect model (teacher 1 and 2), transfer (teacher 1) and assigning tasks and leadership (teacher 3). For teacher 4, no differences were found, other than in fostering interaction and role in assessment. The student observation section of TARE 2.0 revealed statistically significant differences in most behaviors of teacher 1, 2, and 3, except in showing respect (teacher 2 and 3) and helping (teacher 2). For teacher 4, no differences were found other than in engagement and leading.

#### Differences Between Subject Areas

As for the subject areas taught by the four teachers (Table 5), analysis of the 40 sessions (the first four pre-intervention sessions were excluded) in which the TPSR model was applied show statistically significant data (*p* < 0.01) in favor of PE in relation to the following behaviors: respect model, leadership, encouraging and leading. However, History and Spanish Language subject sessions obtained statistically significant data in favor of them in relation to the behavior of interaction, giving choices, transfer, respect (*p* < 0.001), asking for help (*p* < 0.01), and for cooperating (*p* < 0.025).

**Table 5:** Results of TARE 2.0 in teacher and student behaviors.

		PE	History and Spanish	
		Teacher 1 and 4	Teacher 2 and 3	
Category	Variable	*M (SD)*	*M (SD)*	*p*
Teacher	Respect model	3.99 (0.21) 2.36 (1.30)	3.52 (0.62)	0.001^∗∗∗^
	Expectations		2.47 (1.43)	0.082
	Opportunities	2.22 (1.25)	2.13 (1.37)	0.589
	Interaction	2.09 (1.28)	2.49 (1.58)	0.001^∗∗∗^
	Assigning tasks	2.25 (1.37)	2.34 (1.65)	0.190
	Leadership	2.43 (1.77)	0.78 (1.27)	0.001^∗∗∗^
	Giving choices	2.01 (1.23)	2.45 (1.61)	0.001^∗∗∗^
	Assessment	1.18 (1.50)	1.28 (1.50)	0.343
	Transfer	0.28 (0.71)	0.47 (0.87)	0.001^∗∗∗^
	Participation	2.67 (1.18)	2.82 (1.16)	0.066
Student	Engagement	2.25 (0.87)	2.31 (0.91)	0.386
	Respect	2.50 (0.85)	3.26 (0.77)	0.001^∗∗∗^
	Cooperating	1.70 (1.29)	1.94 (1.58)	0.025^∗^
	Encouraging	1.56 (1.51)	0.96 (1.12)	0.001^∗∗∗^
	Helping	1.17 (1.19)	1.12 (1.25)	0.486
	Leading	2.10 (1.82)	0.99 (1.22)	0.001^∗∗∗^
	Expressing	1.83 (1.38)	1.97 (1.54)	0.079
	Help	0.30 (0.70)	0.53 (1.01)	0.010^∗∗^

*Note: p < 0.05 = ^∗^; p < 0.01 = ^∗∗^; p < 0.001 = ^∗∗∗^; Teacher 1 and Teacher 4 = PE teachers; Teacher 2 and Teacher 3 = History and Spanish language teachers.*

## Discussion

The aim of our study was to detect how teachers’ behavior-oriented patterns shifted in response to CPD, thereby reinforcing the implementation of the TPSR (Hellison, 1978) included in the SPM depicted at the beginning of the paper. We obtained significant results in relation to the process of TPSR acquisition in two interesting aspects:

1. After specific teacher training in TPSR (Lee and Choi, 2015) and subsequent assessment, how did each teacher adapt their teaching strategies in their teaching sessions? Analysis results indicate that, for the three teachers who received training in the methodology and for the students, most behaviors reflected improved observation (OSTOR) and statistical significance (TARE) with the application of the TPSR model. Results for the experienced teacher highlighted the application of more diversified TPSR model strategies in the final session and only got better scores in a few strategies because he was already near the maximum possible developmental stage. Overall, the evolution of each teacher tended toward an increase in strategies to encourage responsibility by the students, in line with Escartí et al. (2018), where the TARE behaviors are correlated between the teachers who apply TPSR and student behaviors.
2. Are there differences according to subject area? Regarding the aspect of subject area taught, we would point out that the best results from TARE relating to respect model, leadership, encouraging and leading behaviors appear in PE sessions. We believe this is because PE intrinsically addresses student autonomy (Moreno-Murcia et al., 2008; Aibar et al., 2015). The subject areas of History and Spanish language obtain better results in interaction, giving choices, transfer, respect, asking for help, and cooperation behaviors. This could be, because in these areas teachers promote problem-solving activities oriented to achieving transference to real life (Puigarnau et al., 2016).

Despite these interesting aspects, we have continued to delve into connections with the data obtained by means of the mixed-method approach. Essential data emerge from the T-patterns obtained and from polar coordinate analysis of the observed behaviors of each teacher and their students through comparisons of initial and final sessions. The TARE tool was integrated into this mixed-method approach to detect the responsibility levels established (Hellison and Wright, 2003) and to conduct continued assessment (Hemphill et al., 2015).

### The Evidence of How Teaching Behavior Patterns Shift

We obtained a common and complex T-pattern (Figure 2) that we consider highly relevant because all 21 sets of behaviors included student autonomy. This result reinforces the statement that promoting autonomy attitudes from students is a pillar of the TPSR model (Hellison, 2011). In this common T-pattern, along with the student’s autonomy, another two essential pillars of the TPSR model, and also considered in the TARE tool, appeared: the teaching behaviors of sharing explanations and suggestions (Wright and Craig, 2011; Escartí et al., 2015).

This common T-pattern is nonexistent in the initial sessions of the teachers. Though not linked sequentially, only the behaviors of autonomy, sharing explanations, and suggestions appear in order to create a T-pattern. A proactive change toward TPSR strategies is fully visible in the final sessions of all the teachers when this T-pattern is significant and is even more diversified in the experienced teacher.

### The Evidence of Linkage Between Teacher Strategies and Student Responses

A significant T-pattern and polar coordinate analysis was selected for the initial and final session of each teacher. The contrast of T-patterns and polar coordinate analysis between the initial and final sessions clearly reveals a proactive shift in TPSR implementation. However, in order to show the potential of the mixed-method approach (Anguera et al., 2012, 2014; Castañer et al., 2013b), we decided to explore a new way for an easier understanding of both techniques. We created a new graphical depiction integrating the polar coordinate and T-pattern figures, because the technique of polar coordinate analysis reinforces the results obtained from T-pattern analysis. While T-pattern analysis reveals how behaviors change over time, polar coordinates detect which behaviors are mutually activated or inhibited (Arias-Pujol and Anguera, 2017). The integrated figures show that all teachers experienced a considerable shift in their teaching strategies from directive and controlling intervention, with negative assessments that generate reproduction responses from the students, toward participatory intervention that promotes autonomy responses from the students. The three methodological tools used in this study highlight the same process of pedagogical optimization that promotes student involvement and responsibility (Lorente and Kirk, 2016; Prat et al., 2019a,b).

## Conclusion

The mixed-method approach followed in the current study reveals the benefits that can be achieved with TPSR in an educational context for the improvement of values, as identified by several studies such as that by Pozo et al. (2016). Moreover, TPSR has been shown to be appropriate for facilitating CPD for teachers (Hemphill et al., 2015). Finally, this type of methodology indicates that TPSR implementation is possible for PE as well as other school subjects (Escartí et al., 2018), thereby enabling the teacher to achieve improved behavior interaction and assessment in class and providing students with better opportunities to acquire educational values such as engagement and leading. This study leads us to conclude that social responsibility behavior changes in students (Lorente and Kirk, 2016), based on the TPSR model, provide a favorable framework for activating encouraging and leading behaviors in PE and respect, cooperating and asking for help behaviors in other subject areas. Therefore, the ongoing and continuous application of these educational and pedagogical intervention strategies to educational programs promotes responsibility and autonomy that are a primary objective of education (Belando et al., 2012).

### Prospective Future Lines

This study a systematic method for objectively analyzing the process of teacher optimization in TPSR. We therefore believe that it can serve as a reference framework for studies focusing on the essentials of education:

1. for teachers:
  a. *Enacting action research* that focuses on understanding educational environments in order to innovate and optimize the quality of education linked to pre-service teachers (PSTs). It provides a cyclical and systematic approach to problem-solving that encourages teachers to solve their educational problems through reflection-action (Gibbs et al., 2016);
  b. *Optimizing communicative competencies of teachers*. High communicative competency in the teacher will result in a higher quality of interaction for the student because the processes of teaching and learning are essentially communicative processes (Barbat, 2008; Castañer et al., 2013a). We believe that OSTOR, the observational instrument of this study, could be extended by means of existing specialized observational systems, such as SOCIN and SOPROX, to detect kinesic and proxemic behaviors, respectively (Castañer et al., 2013a, 2016b).
2 for students:
  a. Strengthening self-esteem because it is an integrated set of cognitive, affective, and attitudinal factors that also highlight effectiveness. Therefore, the criteria contained in the TARE could be expanded to include studies that point to reinforcing student self-esteem (Legault et al., 2006).
  b. The acquisition of autonomy and responsibility in education could be framed within the Self-Determination Theory (STD, Deci and Ryan, 2000, 2012), as stated in previous studies (Puigarnau et al., 2016). This theory claims that there are three basic psychological needs (competence, autonomy, and relatedness), the satisfaction of which increases intrinsic motivation and personal well-being.

In sum, as Tissington and Senior (2017) point out, carrying out pedagogical research is essential for effective learning. We believe our study offers a systematized observational tool and two specific techniques that can enhance pedagogical research.

## Ethics Statement

The study was carried out in accordance with the Declaration of Helsinki and was accepted and verified by the Ethics Committee of the University of Murcia, Spain (ID 1685/2017). As the observational methodology we use is always conducted over natural context, we observed the natural development of the scholar sessions, therefore written informed parental consent was not obtained for the purposes of research participation and was not required as per applicable institutional and national guidelines. Regarding video recording, the institution has a consent form about image privacy that parents of students enrolling at the school are required to sign. All four teachers signed a consent form in order to participate in the research study, the consent obtained was both written and informed.

## Author Contributions

MC, OC, and AV-V developed the project, supervised the design of the study, the method section, and the drafting of the manuscript. DMS was responsible for the review of the literature. OC, AV-V, DMS, QP, and MC were responsible for the critical revision of the content. MC, AV-V, and OC were responsible for the drafting of the manuscript. OC, QP, and DMS collected and codified the data. All the authors approved the final, submitted version of the manuscript.

## Conflict of Interest Statement

The authors declare that the research was conducted in the absence of any commercial or financial relationships that could be construed as a potential conflict of interest.

## References

[B1] Aibar A. Julián J. A. Murillo B. García-González L. Estada S. Bois J. (2015). Physical activity and autonomy support: the PE teacher’s role. *Rev. Psicol. Deporte* 24 155–161.

[B2] Alves Franco S. C. da Costa Simoes V. A. Castañer M. Fernandes Rodrigues J. J. Anguera M. T. (2013). Fitness instructor behaviour: triangulation of participant perceptions, instructor self-perception and observed behaviour. *Rev. Psicol. Deporte* 22 321–329.

[B3] Anguera M. T. (1997). “From prospective patterns in behavior to joint analysis with a retrospective perspective,” in *Colloque sur Invitation “Méthodologie d’Analyse des Interactions Sociales”*, eds Arnau J. Anguera M. T. Gómez J. (Paris: Université de la Sorbona).

[B4] Anguera M. T. (2003). “Observational methods (General)”, in *Encyclopedia of Psychological Assessment* Vol. 2 ed. Fernández-Ballesteros R. (London: Sage), 632–637. 10.4135/9780857025753.n136

[B5] Anguera M. T. Camerino O. Castañer M. (2012). “Mixed methods procedures and designs for research on sport, physical education and dance,” in *Mixed Methods Research in the Movement Sciences: Cases in Sport, Physical Education and Dance*, eds Camerino O. Castañer M. Anguera M. T. (London: Routledge), 3–27.

[B6] Anguera M. T. Camerino O. Castañer M. Sánchez-Algarra P. (2014). Mixed methods research in relation to physical activity and sport. *Rev. Psicol. Deporte* 23 123–130.

[B7] Anguera M. T. Camerino O. Castañer M. Sánchez-Algarra P. Onwuegbuzie A. (2017). Sports sciences: moving forward in mixed methods research and proposals for achieving quantitative and qualitative symmetry. *Front. Psychol.* 8:2196. 10.3389/fpsyg.2017.02196 29312061 PMC5742273

[B8] Arias-Pujol E. Anguera M. T. (2017). Observation of interactions in adolescent group therapy: a mixed methods study. *Front. Psychol.* 8:1188. 10.3389/fpsyg.2017.01188 28790937 PMC5522846

[B9] Armour K. Quennerstedt M. Chambers F. Makopoulou K. (2017). What is ‘effective’CPD for contemporary physical education teachers? A Deweyan framework. *Sports Educ. Soc.* 22 799–811. 10.1080/13573322.2015.1083000

[B10] Bandura A. (1977). *Social Learning Theory.* Englewood Cliffs, NJ: Prentice-Hall.

[B11] Barbat B. E. (2008). E-Maieutics. Rationale and approach. *Int. J. Comput. Commun.* 3 40–54.

[B12] Belando N. Ferriz-Morell R. Moreno-Murcia J. A. (2012). Proposal of a model for personal and social improvement through the promotion of responsibility for physical and sporting activity. *Rev. Int. Cienc. Deporte* 8 202–222. 10.5232/ricyde2012.02902

[B13] Burgoon J. K. Wilson D. Hass M. Schuetzlr R. (2016). “Interactive deception in group decision-making: new insights from communication pattern analysis,” in *Discovering Hidden Temporal Patterns in Behavior and Interactions: T-Pattern Detection and Analysis with THEME*, eds Magnusson M. S. Burgoon J. K. Casarrubea M. McNeill D. (New York, NY: Springer), 37–61.

[B14] Camerino O. Castañer M. Anguera M. T. (2012). *Mixed Methods Research in the Movement Sciences: Cases in Sport, Physical Education and Dance.* Abingdon: Routledge.

[B15] Casarrubea M. Jonsson G. K. Faulisi F. Sorbera F. Di Giovanni G. Benigno A. (2015). T-pattern analysis for the study of temporal structure of animal and human behavior: a comprehensive review. *J. Neurosci. Methods* 239 34–46. 10.1016/j.jneumeth.2014.09.024 25280983

[B16] Casarrubea M. Magnusson M. S. Anguera M. T. Jonsson G. K. Castañer M. Santangelo A. (2018). T-pattern detection and analysis for the discovery of hidden features of behaviour. *J. Neurosci. Methods* 310 24–32. 10.1016/j.jneumeth.2018.06.013 29935197

[B17] Castañer M. Barreira D. Camerino O. Anguera M. T. Fernandes T. Hileno R. (2017). Mastery in goal scoring, T-pattern detection and polar coordinate analysis of motor skills used by lionel messi and cristiano ronaldo. *Front. Psychol.* 8:741. 10.3389/fpsyg.2017.00741 28553245 PMC5427849

[B18] Castañer M. Camerino O. Anguera M. T. Jonsson G. K. (2010). Observing the paraverbal communicative style of expert and novice PE teachers by means of SOCOP: a sequential análisis. *Procedia Soc. Behv. Sci.* 2 5162–5167. 10.1016/j.sbspro.2010.03.839

[B19] Castañer M. Camerino O. Anguera M. T. Jonsson G. K. (2013a). Kinesics and proxemics communication of expert and novice PE teachers. *Qual. Quant.* 47 1813–1829. 10.1007/s11135-011-9628-5

[B20] Castañer M. Camerino O. Anguera M. T. (2013b). Métodos Mixtos en la investigación de las Ciencias de la Actividad Física y el Deporte [Mixed Methods in the Research of Sciences of Physical Activity and Sport]. *Apunts. Educación Física y Deportes* 112 11–20. 10.5672/apunts.2014-0983.es.(2013/2).112.01

[B21] Castañer M. Camerino O. Landry P. Parés N. (2016a). Quality of physical activity of children in exergames: sequential body movement analysis and its implications for interaction design. *Int. J. Hum. Comput. Stud.* 96 67–78. 10.1016/j.ijhcs.2016.07.007

[B22] Castañer M. Camerino O. Anguera M. T. Jonsson G. K. (2016b). “Paraverbal Communicative Teaching T-Patterns Using SOCIN and SOPROX Observational Systems,” in *Discovering Hidden Temporal Patterns in Behavior and Interaction*, eds Magnusson M. S. Burgoon J. K. Casarrubea M. (New York, NY: Springer), 83–100. 10.1007/978-1-4939-3249-8

[B23] Castañer M. Camerino O. Parés N. Landry P. (2011). Fostering body movement in children through an exertion interface as an educational tool. *Procedia Soc. Behv. Sci.* 28 236–240. 10.1016/j.sbspro.2011.11.046

[B24] Castañer M. Franco S. Rodrigues J. Miguel C. (2012). “Optimizing verbal and nonverbal communication in PE teachers, instructors and sport coaches,” in *Mixed Methods Research in the Movement Sciences: Cases in Sport, Physical Education and Dance*, eds Camerino O. Castañer M. Anguera M. T. (London: Routledge), 177–214.

[B25] Cecchini J. Montero J. Alonso A. Izquierdo M. Contreras O. (2007). Effects of personal and social responsibility on fair play in sports and self-control in school-aged youths. *Eur. J. Sport Sci.* 7 203–211. 10.1080/17461390701718497

[B26] Creswell J. W. (2003). *Research Design: Qualitative, Quantitative, and Mixed Methods Approaches*, 2nd Edn. Thousand Oaks, CA: Sage.

[B27] Creswell J. W. (2015). *A Concise Introduction to Mixed Methods Research.* Thousand Oaks, CA: Sage.

[B28] Cutforth N. Puckett K. (1999). An investigation into the organization, challenges, and impact of an urban apprentice teacher program. *Urban Rev.* 31 153–172.

[B29] DeBusk M. Hellison D. (1989). Implementing a Physical Education Self-Responsibility Model for delinquency-prone youth. *J. Teach. Phys. Educ.* 104–112. 10.1123/jtpe.8.2.104

[B30] Deci E. L. Ryan R. M. (2000). The “what” and “why” of goal pursuits: human needs and the self-determination of behavior. *Psychol. Inq.* 11 227–268. 10.1207/S15327965PLI1104_01 27055568

[B31] Deci E. L. Ryan R. M. (2012). “Self-determination theory,” in *Handbook of Theories of Social Psychology*, eds P. A. M. Van Lange Kruglanski A. W. Higgins E. T. (Thousand Oaks, CA: Sage), 416–437. 10.4135/9781446201022

[B32] Escartí A. Gutiérrez M. Pascual C. Marín D. (2010a). Application of Hellison’s Teaching Personal and Social Responsibility model in physical education to improve self-efficacy for adolescents at risk of dropping-out of school. *Span. J. Psychol.* 13 667–676. 10.1017/S113874160000233X 20977016

[B33] Escartí A. Gutiérrez M. Pascual C. Llopis R. (2010b). Implementation of the personal and social responsibility model to improve self-efficacy during physical education classes for primary school children. *Int. J. Psychol. Psychol. Therapy* 10 387–402. 10.4100/jhse.2012.82.10

[B34] Escartí A. Gutiérrez M. Pascual C. Wright P. (2013). Observación de las estrategias que emplean los profesores de educación física para enseñar la responsabilidad (TARE) [Observation of the strategies that physical education teachers use to teach responsibility]. *Rev. Psicol. Deporte* 22 159–166. 5052075

[B35] Escartí A. Llopis-Goig R. Wright P. M. (2018). Assessing the Implementation Fidelity of a School-based Teaching Personal and Social Responsibility Program in Physical Education and Other Subject Areas. *J. Teach. Phys. Educ.* 37 12–23. 10.1123/jtpe.2016-0200

[B36] Escartí A. Wright P. Pascual C. Gutiérrez M. (2015). Tool for assessing responsibility-based education (TARE) 2.0: instrument revisions, inter-rater reliability, and correlations between observed teaching strategies and student behaviors. *Univ. J. Psychol.* 3 55–63. 10.13189/ujp.2015.030205

[B37] Fernández-Hermógenes D. Camerino O. Garcia de Alcaraz A. (2017). Set-piece Offensive Plays in Soccer. *Apunts. Educación Física y Deportes* 129 64–77. 10.5672/apunts.2014-0983.es.(2017/3).129.06

[B38] Gabin B. Camerino O. Anguera M. T. Castañer M. (2012). Lince: multiplatform sport analysis software. *Procedia Soc. Behav. Sci.* 46 4692–4694. 10.1016/j.sbspro.2012.06.320

[B39] García-López L. M. Gutiérrez D. González-Víllora S. Valero A. (2012). Cambios en la empatía, la asertividad y las relaciones sociales por la aplicación del modelo de instrucción educación deportiva [Changes in empathy, assertiveness and social relations due to the implementation of the sport education model]. *Rev. Psicol. Deporte* 22 321–330.

[B40] Gibbs P. Cartney P. Wilkinson K. Parkinson J. Cunningham S. James-Reynolds C. (2016). Literature review on the use of action research in higher education. *Educ. Act. Res.* 25 3–22. 10.1080/09650792.2015.1124046

[B41] Goodyear V. A. Casey A. Kirk D. (2014). Tweet me, message me, like me: using social media to facilitate pedagogical change within an emerging community of practice. *Sport Educ. Soc.* 19 927–943. 10.1080/13573322.2013.858624

[B42] Gordon B. (2010). An examination of the responsibility model in a New Zealand secondary school physical education program. *J. Teach. Phys. Educ.* 29 21–37. 10.1123/jtpe.29.1.21

[B43] Hastie P. A. Casey A. (2014). Fidelity in models-based practice research in sport pedagogy: a guide for future investigations. *J. Teach. Phys. Educ.* 33 422–431. 10.1123/jtpe.2013-0141

[B44] Hellison D. (1978). *Beyond Balls and Bats: Allienated (and Other) Youth in the Gym.* Washington DC: AAHPER.

[B45] Hellison D. (1985). *Goals and Strategies for Teaching Physical Education.* Champaign, IL: Human Kinetics.

[B46] Hellison D. (2011). *Teaching Responsibility through Physical Activity*, 3rd Edn. Champaign, IL: Human Kinetics.

[B47] Hellison D. Wright P. M. (2003). Retention in an urban extended day program: a process-based assessment. *J. Teach. Phys. Educ.* 22 369–381. 10.1123/jtpe.22.4.369

[B48] Hemphill M. A. Templin T. J. Wright P. M. (2015). Implementation and outcomes of a responsibility-based continuing professional development protocol in physical education. *Sport Educ. Soc.* 20 398–419. 10.1080/13573322.2012.761966

[B49] Hernández-Mendo A. Castellano J. Camerino O. Jonsson G. Blanco-Villaseñor A. (2014). Observational software, data quality control and data analysis. *Rev. Psicol. Deporte* 23 111–121.

[B50] Ivy V. N. Richards K. A. R. Lawson M. A. Alameda-Lawson T. (2018). Lessons learned from an after-school program: building personal and social responsibility. *J. Youth Dev.* 13 162–175. 10.5195/jyd.2018.606

[B51] Kluge A. (2008). What you train is what you get? Task requirements and training methods in complex problem-solving. *Comput. Hum. Behav.* 24 284–308. 10.1016/j.chb.2007.01.013

[B52] Lee O. Choi E. (2015). The influence of professional development on teachers’ implementation of the Teaching Personal and Social Responsibility model. *J. Teach. Phys. Educ.* 34 603–625. 10.1123/jtpe.2013-0223

[B53] Legault L. Green-Demers I. Pelletier L. (2006). Why do high school students lack motivation in the classroom? Toward an understanding of academic amotivation and the role of social support. *J. Educ. Phychol.* 98 567–582. 10.1037/0022-0663.98.3.567

[B54] Likfa B. (1990). Hiding beneath the Stairwell-A dropout prevention program for Hispanic youth. *J. Phys. Educ. Recreat. Dance* 61 40–41. 10.1080/07303084.1990.10604548

[B55] LOMCE (2013). *Ley Orgánica Para la Mejora de la Calidad Educativa. [Organic Law for the Improvement of Educational Quality], 8/2013.* Available at: https://www.boe.es/eli/es/lo/2013/12/09/8/con (accessed December 10, 2013).

[B56] López Jiménez J. Valero-Valenzuela A. Anguera M. T. Díaz Suárez A. (2016). Disruptive behavior among elementary students in physical education. *SpringerPlus* 5:1154. 10.1186/s40064-016-2764-6 27504252 PMC4958097

[B57] Lorente E. Kirk D. (2016). Student teachers’ understanding and application of assessment for learning during a physical education teacher education course. *Eur. Phys. Educ. Rev.* 22 65–81. 10.1177/1356336X15590352

[B58] Lozano D. Camerino O. Hileno R. (2016). Dynamic Offensive Interaction in High Performance Handball. *Apunts. Educación Física y Deportes* 125 90–110. 10.5672/apunts.2014-0983.es.(2016/3).125.08

[B59] Magnusson M. S. Burgoon J. K. Casarrubea M. (eds) (2016). *Discovering Hidden Temporal Patterns in Behavior and Interaction: T-Pattern Detection and Analysis with THEME.* New York, NY: Springer-Verlag.

[B60] Moreno-Murcia J. A. Parra N. González-Cutre D. (2008). Influencia del apoyo a la autonomía, las metas sociales y la relación con los demás sobre la desmotivación en educación física.[Influence of autonomy support, social goals and relatedness on amotivation in physical education]. *Psicothema* 20 636–641. 18940062

[B61] O’Cathain A. Murphy E. Nicholl J. (2010). Three techniques for integrating data in mixed methods studies. *Br. Med. J.* 341 c4587. 10.1136/bmj.c4587 20851841

[B62] Pérez-Tejera F. Valera S. Anguera M. T. (2018). Using systematic observation and polar coordinates analysis to assess gender-based differences in park use in Barcelona. *Front. Psychol.* 9:2299. 10.3389/fpsyg.2018.02299 30542307 PMC6277760

[B63] Piaget J. Cook M. (1952). *The Origins of Intelligence in Children.* New York, NY: International University Press.

[B64] Pozo P. Grao-Cruces A. Pérez-Ordás R. (2016). Teaching personal and social responsibility model-based programmes in physical education. *Eur. Phys. Educ. Rev.* 24 56–75. 10.1177/1356336X16664749

[B65] Prat Q. Andueza J. Echávarri B. Camerino O. Fernandes T. Castañer M. (2019a). A mixed methods design to detect adolescent and young adults’ impulsiveness on decision-making and motor performance. *Front. Psychol.* 10:1072. 10.3389/fpsyg.2019.01072 31178778 PMC6543009

[B66] Prat Q. Camerino O. Castañer M. Andueza J. Puigarnau S. (2019b). The personal and social responsibility model to enhance innovation in physical education. *Apunts. Educación Física y Deportes* 136 83–99. 10.5672/apunts.2014-0983.es.(2019/2).136.06

[B67] Puigarnau S. Camerino O. Castañer M. Prat Q. Anguera M. T. (2016). The importance of the support to the autonomy in practitioners of sports centers and fitness to increase its motivation and adhesion. *Rev. Int. Cienc. Deporte* 43 48–64. 10.5232/ricyde2016.04303

[B68] Rodríguez-Dorta M. Borges A. (2017). Behavioral patterns in special education, good teaching practices. *Front. Psychol.* 8:631. 10.3389/fpsyg.2017.00631 28512437 PMC5412478

[B69] Sackett G. P. (1980). “Lag sequential analysis as a data reduction technique in social interaction research”, in *Exceptional Infant: Psychosocial Risks in Infant-Environment Transactions* Vol. 4 eds Sawin D. B. Hawkins R. C. Walker L. O. Penticuff J. H. (New York, NY: Brunner-Mazel), 300–340.

[B70] Sáenz A. Gimeno F. Gutiérrez H. Garay B. (2012). Prevención de la agresividad y la violencia en el deporte en edad escolar: Un estudio de revisión. *Cuadernos de Psicologa del Deporte* 12 57–72. 10.4321/S1578-84232012000200007

[B71] Sánchez-Alcaraz B. J. Cañadas M. Valero A. Gómez A. Funes A. (2019). Results, difficulties and improvements in the model of personal and social responsibility. *Apunts. Educación Física y Deportes* 136 62–82. 10.5672/apunts.2014-0983.es.(2019/2).136.05

[B72] Smithikrai C. (2013). “The mediating roles of academic stress and life satisfaction in the relationship between personal responsibility and academic performance,” in *Innovation, Communication and Engineering*, eds Lumban F. Kadry S. Taylor M. Shen P. (London: Taylor & Francis Group), 383–387.

[B73] Soto A. Camerino O. Iglesias X. Anguera M. T. Castañer M. (2019). LINCE PLUS: Research software for behavior video analysis. *Apunts. Educación Física y Deportes* 137 149–153. 10.5672/apunts.2014-0983.es.(2019/3).137.11

[B74] Tissington P. Senior C. (2017). Research activity and the new pedagogy: why carrying out research is essential for effective learning. *Front. Psychol.* 8:1838. 10.3389/fpsyg.2017.01838 29097990 PMC5654386

[B75] Torrents C. Castañer M. Dinusova M. Anguera M. T. (2013). Dance divergently in physical education: teaching using open-ended questions, metaphors, and models. *Res. Danc. Educ.* 2 104–119. 10.1080/14647893.2012.712100

[B76] Vygotsky L. (1978). *Mind in Society: The Development of Psychological Processes.* Cambridge, MA: Harvard University Press.

[B77] Waring M. Evans C. (2015). *Understanding Pedagogy: Developing a Critical Approach to Teaching and Learning.* Abingdon: Routledge.

[B78] Wright P. M. Craig M. W. (2011). Tool for assessing responsibility-based education (TARE): Instrument development, content validity, and inter-rater reliability. *Measure. Phys. Educ. Exerc. Sci.* 15 204–219.

[B79] Wright P. M. Li W. Ding S. Pickering M. (2010). Integrating a personal and social responsibility program into a wellness course for urban high school students: assessing implementation and educational outcomes. *Sport Educ. Soc.* 15 277–298. 10.1080/13573322.2010.493309

